# Diallyl Disulfide Suppresses Inflammatory and Oxidative Machineries following Carrageenan Injection-Induced Paw Edema in Mice

**DOI:** 10.1155/2020/8508906

**Published:** 2020-04-15

**Authors:** Hongxing Zhang, Chi Shang, Zhao Tian, Hatem K. Amin, Rami B. Kassab, Ahmed E. Abdel Moneim, Yingang Zhang

**Affiliations:** ^1^Department of Orthopaedic Surgery, The First Affiliated Hospital of Xi'an Jiaotong University, No. 277 West Yanta Road, Yanta District, Xi'an, Shaanxi 710061, China; ^2^Department of Hand Surgery, Xi'an Honghui Hospital, No. 555 Youyi East Road, Beilin District, Xi'an, Shaanxi 710054, China; ^3^Department of Biochemistry and Molecular Biology, Faculty of Pharmacy, Helwan University, Cairo 11795, Egypt; ^4^Zoology and Entomology Department, Faculty of Science, Helwan University, Cairo 11795, Egypt; ^5^Biology Department, Faculty of Science and Arts, Al Baha University, Al Makhwah Branch, Saudi Arabia

## Abstract

Diallyl disulfide (DADS) is the major organosulfur constituent in garlic, with a variety of pharmacological activities including antioxidant and anti-inflammatory. Here, we examined the potential antiedematous impact of DADS- versus carrageenan-mediated paw edema in mice. Carrageenan injection potentiated an inflammatory reaction as presented by the elevated serological C-reactive protein (CRP) levels and transcription of tumor necrosis factor-alpha (TNF-*α*, Tnf*α*), interleukin-1 beta (IL-1*β*, Il1b), interleukin-2 (IL-2, Il2), inducible nitric oxide synthase (iNOS), nitric oxide (NO), cyclooxygenase-2 (COX-2, Ptgs2), prostaglandin E2 (PGE2), monocyte chemoattractant protein-1 (MCP-1, Ccl1), nuclear factor kappa B (NF-*κ*B), and myeloperoxidase (MPO) activity, while interleukin-10 (IL-10) was declined in the injured paw tissue. Additionally, carrageenan elevated lipid peroxidation in terms of malondialdehyde (MDA) and decreased glutathione content (GSH). Remarkably, DADS was found to inhibit the inflammatory signaling, suppressed the developed oxidative damage, and protected the histopathological alterations in the inflamed paw tissue in response to carrageenan injection. Our findings suggest that DADS could be used as an alternative therapy used to alleviate the pathophysiological changes associated with the genesis of paw edema through its potent anti-inflammatory and antioxidant impacts.

## 1. Introduction

Inflammation is a protective physiological defense mechanism provided by the immune system in response to toxins, infectious pathogens, and local injury [1]. It is commonly characterized by redness and swelling of tissues and is also associated with hyperthermia and pain. Inflammation has been correlated with the progression of several diseases including cancer, cardiovascular, and aging-related disorders [2]. Acute inflammatory response is considered a part of innate immunity which entails the recruit of immune cells including macrophages, lymphocytes, neutrophils, mast, and dendritic cells and also includes the generation of reactive oxygen species (ROS) and different inflammatory mediators in order to limit the spread of the cellular injury [3]. Carrageenan-induced paw edema is used as a model for acute inflammation in experimental animals to explore the potential anti-inflammatory effect of novel agents [2]. Once injected, carrageenan enhances the production and release of several inflammatory mediators including histamine, prostaglandins, neutrophil-derived free radicals, nitric oxide, and mainly the proinflammatory cytokines [2]. The application of current anti-inflammation drugs including nonsteroid anti-inflammatory drugs, analgesics, and corticosteroids has been associated with several adverse reactions. Hence, searching for new anti-inflammatory agents with minimum side effects is mandatory [4]. Medicinal plants are currently used in a wide range as alternative therapeutic agents to prevent or treat different health problems including inflammation and its related disorders [5]. Agents that suppress cyclooxygenase enzymes, inducible nitric oxide synthase expressions, and chemokines have been suggested to minimize the inflammatory reactions and its consequences in carrageenan-induced inflammation [2].

Garlic (*Allium sativum* L.) has been widely used in dietary and medicinal purposes due to its various bioactive ingredients, including organosulfur compounds (alliin, allicin, diallyl sulfide, diallyl disulfide, diallyl trisulfide, E/Z-ajoene, and S-allyl-cysteine), saponins (desgalactotigonin-rhamnose, proto-desgalactotigonin, proto-desgalactotigonin-rhamnose, voghieroside D1, sativoside B1-rhamnose, and sativoside R1), phenolic compounds (*β*-resorcylic acid, pyrogallol, gallic acid, rutin, protocatechuic acid, and quercetin), and polysaccharides (fructose and glucose) [6]. The anti-inflammatory activity of the organosulfur molecules found in garlic has been studied. Quintero-Fabian et al. [7] showed that alliin decreased the level and expression of interleukin-6, monocyte chemoattractant protein-1, and early growth receptor-1. In addition, alliin inhibited the phosphorylation of ERK1/2 which plays a crucial role in the development of inflammation in lipopolysaccharide- (LPS-) stimulated 3T3-L1 adipocytes. Moreover, allicin attenuated markedly the inflammatory responses and oxidative damage in human umbilical vein endothelial cells upon LPS stimulation through inhibiting endothelial cell adhesion and tumor necrosis factor-*α* (TNF-*α*) and interleukin-8 release along with the upregulation of nuclear factor erythroid 2-related factor 2 (Nrf2) [8]. Meanwhile, diallyl sulfide administration along with carbon tetrachloride showed antioxidant and anti-inflammatory capacity through enhancing the Nrf2 pathway and reducing production of interleukin-1*β* (IL-1 *β*) and TNF-*α* and deactivating nuclear factor kappa B (NF-*κ*B). Diallyl sulfide (DADS) like the major organosulfur compounds in garlic exhibited various biological and pharmacological applications such as antioxidant, anti-inflammatory, and immunomodulatory activities [9, 10]. The antioxidant capacity of DADS is due to its ability to scavenge ROS and enhancing the activity of heme oxygenase-1 (HO-1) and Nrf2 signaling [11]. In addition, DADS exerts its anti-inflammatory and immunomodulatory roles through suppressing the translocation of NF-*κ*B and downregulating the expression of proinflammatory cytokines [10]. Although the anti-inflammatory activity of garlic containing organosulfur compounds has been studied previously, the potential anti-inflammatory activity of DADS against carrageenan-induced paw edema has not been explored yet. Hence, the current experiment was undertaken to explore the potential protective role of DADS against carrageenan-induced paw edema in mice through focusing on the levels and expression of different inflammatory mediators along with malondialdehyde (MDA) and glutathione (GSH) content in the inflamed paw skin.

## 2. Materials and Methods

### 2.1. Chemicals

Diallyl disulfide (DADS, CAS number: 2179-57-9, 98% purity) was sourced from Sigma-Aldrich Chemical Co. (St. Louis, MO, USA), and all other applied chemicals were of analytical grade.

## 3. Carrageenan-Induced Paw Edema

Male Swiss mice (20–25 g) were divided into four groups: control, carrageenan, DADS (100 mg/kg), and diclofenac as a reference drug (20 mg/kg) according to Gupta et al. [12]. The chosen dose of DADS was selected based on preliminary experiment using 50, 75, and 100 mg/kg. The higher dose was found to be the best dose among these three doses in treating paw edema induced by carrageenan which was also in agreement with a previous report [13]. DADS and diclofenac were dissolved in 0.9% NaCl and gavaged to the mice for 5 consecutive days before carrageenan injection. Paw edema was induced by injecting 0.1 mL of 1% *w*/*v* carrageenan suspended in saline into the subplantar tissues of the left hind paw of each mouse. The volume of the paw was measured with a Vernier caliper (LETICA Scientific Instruments, Barcelona, Spain) immediately prior to carrageenan injection and again at 2, 4, 6, and 8 h after injection. The data were presented as the variation in the paw volume (mL) and were compared to the right hind paw of the same mouse. At 8 h, animals were euthanized and paw skin samples were collected and divided into two samples. One sample was homogenized immediately to yield 50% (*w*/*v*) homogenate in ice-cold medium containing 50 mM Tris-HCl (pH 7.4) and centrifuged at 500 × *g* for 10 min at 4°C. The supernatant was used for the various biochemical assays, while the second sample was used for the histological or molecular studies. The animal care procedures agreed with the National Institutes of Health (NIH) Guidelines for the Care and Use of Laboratory Animals 8th edition and were approved by the Institutional Animal Ethics Committee guidelines for Laboratory Animal Care at Zoology department, Faculty of Science, Helwan University (Approval Number: HU2017/Z/04).

### 3.1. Cytokine and Inflammatory Mediator Analyses

Levels of tumor necrosis factor alpha (TNF-*α*; cat. no: CSB-E04741m), interlukin-1 beta (IL-1*β*; cat. no: CSB-E04621m), interlukin-2 (IL-2; cat. no: CSB-E04627m), interlukin-10 (IL-10; cat. no: CSB-E04594m), cyclooxygenase-2 (COX-2; cat. no: CSB-E12910m), prostaglandin E2 (PGE2; cat. no: CSB-PA040059), and monocyte chemoattractant protein-1 (MCP-1; cat. no: CSB-E07430m) were assessed in a paw supernatant by ELISA using anti-mouse antibody (CUSABIO Life Sciences, Wuhan, China) based on the manufacturer's instructions.

### 3.2. Myeloperoxidase (MPO) Activity

MPO activity was assayed according to the modified protocol described by Bradley et al. [14]. After three freeze-thaw cycles of the homogenate and centrifugation at 15,000 × *g* for 10 min at 4°C, MPO activity was estimated by mixing 200 *μ*L of the paw supernatant with 2.8 mL of 50 mM phosphate buffer (pH 6.0) and 1 mL of 1.67 mM *o*-dianisidine hydrochloride containing 0.0005% (*v*/*v*) H_2_O_2_. The change in the absorbance at 450 nm was recorded, and MPO activity was presented as U/mg protein.

### 3.3. Measurement of Nitrite/Nitrate Levels

The assay for analyzing the nitrite/nitrate levels in paw supernatant was performed according to the method of Green et al. [15] by adding the Griess reagent (a mixture of naphthylene diamine dihydrochloride (0.1%) and sulfanilamide (1% in 5% H_3_PO_4_)) for 10 min in dark at 30°C, and the absorbance of the bright reddish-purple azo dye was determined at 540 nm.

### 3.4. Estimation of Lipid Peroxidation

Lipid peroxidation (LPO) in the paw supernatant was estimated using 1 mL of 0.67% thiobarbituric acid and 1 mL of 10% trichloroacetic acid in a boiling water bath for 30 min. Thiobarbituric acid reactive substances were determined by absorbance at 535 nm and expressed as the developed amount of malondialdehyde (MDA) [16].

### 3.5. Determination of Glutathione

The level of glutathione (GSH) in the paw tissue was determined by the reduction of 5,5′-dithiobis (2-nitrobenzoic acid) (Ellman's reagent) with GSH to form a yellow compound. The amount of reduced chromogen is directly proportional to the GSH content and its absorbance can be estimated at 405 nm [17].

### 3.6. Quantitative Real-Time PCR

Total RNA was isolated, and first-strand cDNA was synthesized according to the manufacturer's protocol. The mRNA expression of *Il1b*, *Il2*, *Il10*, *Ptgs2*, *Ccl2*, *Tnfα*, and *Nos2* was estimated using real-time quantitative reverse transcription polymerase chain reaction (qRT-PCR) technique using an Applied Biosystems 7500 Instrument. The thermal conditions for qRT-PCR were denaturated initially at 94°C for 2 min, followed by 40 cycles of 94°C for 30 s and 60°C for 30 s, and a final extension at 72°C for 10 min. After PCR amplification, the *Δ*Ct from three repeated experiments was determined by subtracting the Ct value of the standard gene, glyceraldehyde 3-phosphate dehydrogenase (*Gapdh*), from that of each sample (Ct). The employed primer sequences of different examined genes are provided in Table 1.

### 3.7. Histopathological and Immunohistochemical Analysis

The hind paw skins were fixed in 4% neutral buffered formaldehyde for 24 h. Samples were then paraffinized, sectioned (4–5 *μ*m), and stained with hematoxylin and eosin (H&E) to examine the histopathological changes. Immunoreactivities of inducible nitric oxide synthase (iNOS) and nuclear factor kappa B (NF-*κ*B) profiles were investigated in the paw tissue using purified primary antibodies with avidin-biotin-peroxidase (ABC) and peroxidase substrate (Pierce™ Peroxidase IHC Detection Kit, Thermo Fisher Scientific, CA, USA). Briefly, 0.3% H_2_O_2_ was added to suppress the activity of the endogenous peroxidase. The sections were then incubated with primary antibody overnight at 4°C in a humidified room followed by incubation with biotinylated rabbit anti-mouse secondary antibody (Dako System kit) and avidin-biotin complex (ABC) reagents for 1 h at 30°C in a humidified room. Finally, the specimens were counterstained with hematoxylin, dehydrated, and mounted using Aquatex fluid (Merck KGaA, Darmstadt, Germany). Quantification of immunopositive staining intensity was performed using ImageJ Fiji software (Johannes Schindelin, Albert Cardona, Mark Longair, Benjamin Schmid, and others, https://imagej.net/Fiji/Downloads), version 1.2 (no specific plug-in was applied).

### 3.8. Statistical Analyses

All data are expressed as the mean ± standard deviation (SD). One-way analysis of variance (ANOVA) followed by Duncan's test was utilized to compare the control and treated groups using the statistical package SPSS, version 17.0. A *p* value < 0.05 was considered statistically significant.

## 4. Results

### 4.1. Effects of Diallyl Disulfide on Carrageenan-Induced Paw Edema

**Figure 1:**
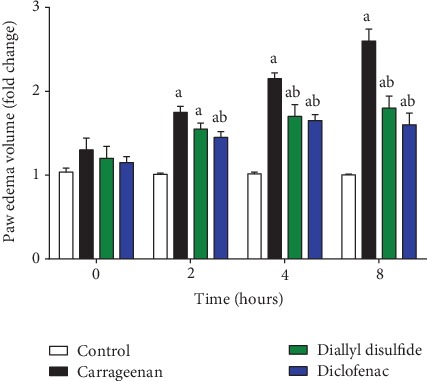
Effect of diallyl disulfide (DADS, 100 mg/kg) or diclofenac (20 mg/kg) on paw edema volume in carrageenan-induced paw edema in mice. Data are represented as mean ± SD (*n* = 7); ^a^*p* < 0.05 indicates a significant change versus the control group; ^b^*p* < 0.05 indicates a significant change versus the carrageenan-injected mice.

Carrageenan injection enhanced the development of the vascular phase of inflammation, resulting in the increased size of edema in the hind paw skin. Interestingly, both diclofenac (20 mg/kg) and DADS administration at a dose 100 mg/kg for five days prior carrageenan injection inhibited significantly (*p* < 0.05) the formed paw edema in a time-dependent effect after 2, 4, and 8 h postcarrageenan injection, with the maximum inhibition noticed following DADS administration after 8 h (-30.76%) when compared to the carrageenan-injected group (Figure 1). The results indicated that the inhibitory action of DADS on the early and late phases of the developed edema.

### 4.2. Effects of Diallyl Disulfide on Blood CRP Level

**Figure 2:**
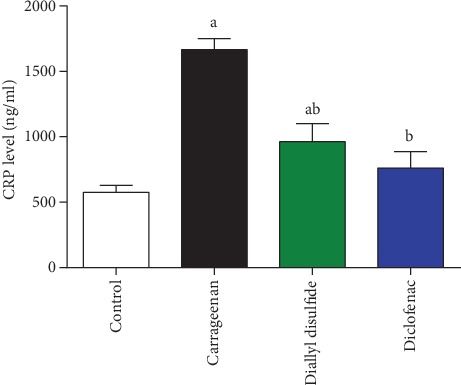
Effect of diallyl disulfide (DADS, 100 mg/kg) or diclofenac (20 mg/kg) on blood C-reactive protein (CRP) in carrageenan-induced paw edema in mice. Data are represented as mean ± SD (*n* = 7); ^a^*p* < 0.05 indicates a significant change versus the control group; ^b^*p* < 0.05 indicates a significant change versus the carrageenan-injected mice.

The blood level of CRP was determined as a vascular inflammatory marker. Carrageenan injection elevated significantly the CRP level as compared to the vehicle control group (Figure 2). DADS administration after carrageenan decreased significantly (*p* < 0.05) the CRP level as compared to the carrageenan-treated mice. The recorded findings indicated that DADS had a potent effect on decreasing the plasma levels of CRP as compared to the used standard drug, diclofenac. Thus, the anti-inflammatory properties of DADS may contribute to the inhibition of edema development.

### 4.3. Effects of Diallyl Disulfide on Carrageenan-Induced Histopathological Changes

**Figure 3:**
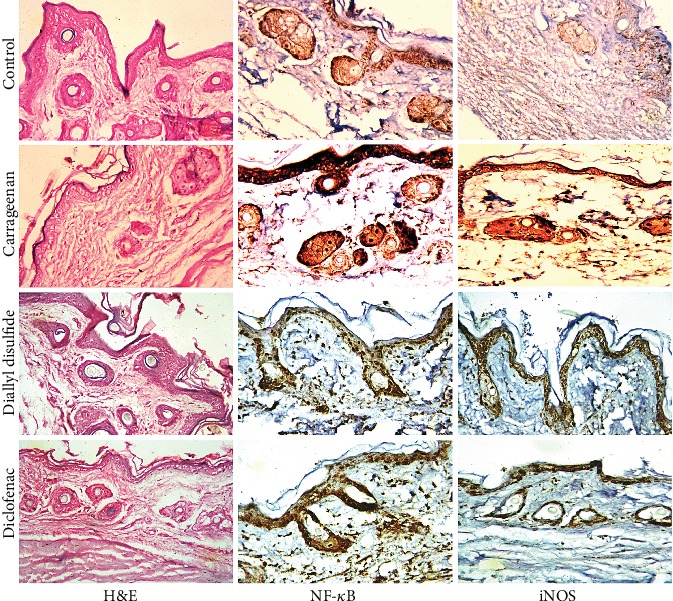
Effect of diallyl disulfide (DADS, 100 mg/kg) or diclofenac (20 mg/kg) on paw skin histology and NF-*κB* and iNOS expression detected by immunohistochemistry following carrageenan injection-induced paw edema in mice, 400x.

As shown in Figure 3, histopathological evaluation of the paw tissue of carrageenan-injected mice revealed epithelial hyperplasia, infiltration of inflammatory cell, and subepidermal edema. These signs of inflammation were largely prevented by DADS. Likewise, the anti-inflammatory edema response evoked by DADS was similar to that exerted by diclofenac treatment.

### 4.4. Effects of Diallyl Disulfide on NF-*κ*B Expression

To elucidate the molecular anti-inflammatory mechanism of DADS, the expression of NF-*κ*B was examined in the paw tissue using a immunohistochemistry technique (Figure 3). NF-*κ*B regulates and controls a variety of inflammatory mediators associated with the development of inflammatory responses. Carrageenan injection was found to activate the expression NF-*κ*B in the inflamed skin tissue as compared to their corresponding control skin tissue. Remarkably, DADS was able to downregulate the activated NF-*κ*B in response to carrageenan injection, which may explain its anti-inflammatory effect. This effect was similar to that offered by the applied standard drug. Quantitative analysis has shown that the mean numbers of NF-*κ*B and iNOS-positive cell intensity were significantly increased in carrageenan-injected mice and decreased by DADS or diclofenac treatment (supplementary data, Figure S1).

### 4.5. Effects of Diallyl Disulfide on Cytokine Secretion

**Figure 4:**
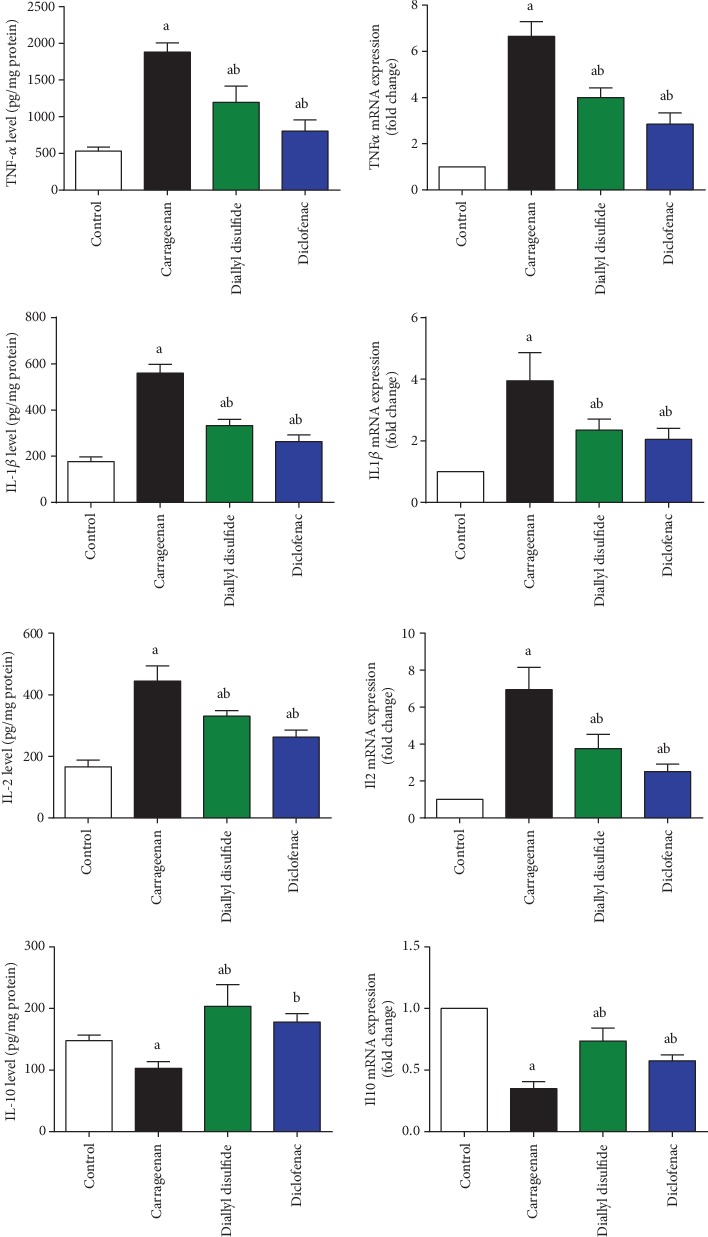
Effect of diallyl disulfide (DADS, 100 mg/kg) or diclofenac (20 mg/kg) on levels and mRNA expression of tumor necrosis factor alpha (TNF-*α*, *Tnfα*), interlukin-1 beta (IL-1*β*, *Il1b*), interlukin-2 (IL-2, *Il2*), and interlukin-10 (IL-10, *Il10*) in carrageenan-induced paw edema in mice. Cytokine level are represented as mean ± SD (*n* = 7). Cytokine mRNA expressions are presented as mean ± SD of triplicate assays and referenced to *Gapdh*. ^a^*p* < 0.05 indicates a significant change versus the control group; ^b^*p* < 0.05 indicates a significant change versus the carrageenan-injected mice.

As shown in Figure 4, carrageenan injection elevated significantly (*p* < 0.05) the release of proinflammatory cytokines (TNF-*α*, IL-1*β*, and IL-2) and upregulated their mRNA expression, while decreased significantly the level and mRNA expression of the anti-inflammatory cytokine (IL-10) when compared to the untreated mice. In contrast, DADS and diclofenac administration showed similar results and decreased the increased levels and expression of proinflammatory cytokines and increased the expression levels of the examined anti-inflammatory cytokine as compared to the carrageenan-injected group.

### 4.6. Effects of Diallyl Disulfide on COX-2 and iNOS Expression and Their Products (PGE2 and NO)

**Figure 5:**
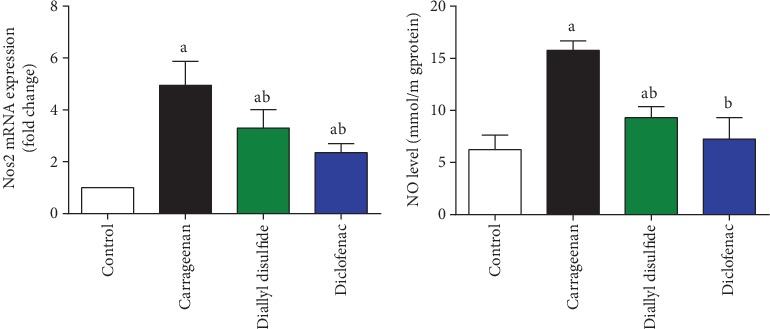
Effect of diallyl disulfide (DADS, 100 mg/kg) or diclofenac (20 mg/kg) on the mRNA expression of inducible nitric oxide synthase (iNOS) using qRT-PCR technique and nitric oxide (NO) level in carrageenan-induced paw edema in mice. ELISA data are represented as mean ± SD (*n* = 7). qRT-PCR data are presented as mean ± SD of triplicate assays and referenced to *Gapdh*. ^a^*p* < 0.05 indicates a significant change versus the control group; ^b^*p* < 0.05 indicates a significant change versus the carrageenan-injected mice.

**Figure 6:**
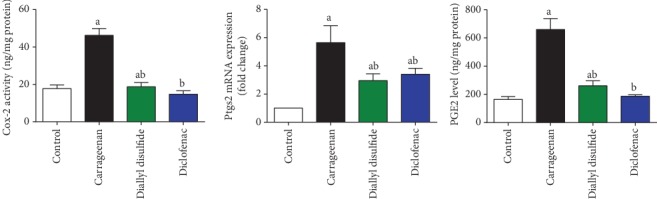
Effect of diallyl disulfide (DADS, 100 mg/kg) or diclofenac (20 mg/kg) on levels and mRNA expression of cyclooxygenase-2 (COX-2, *Ptgs2*) and prostaglandin E2 (PGE2) in carrageenan-induced paw edema in mice. ELISA data are represented as mean ± SD (*n* = 7). qRT-PCR data are presented as mean ± SD of triplicate assays and referenced to *Gapdh*. ^a^*p* < 0.05 indicates a significant change versus the control group; ^b^*p* < 0.05 indicates a significant change versus the carrageenan-injected mice.

Immunohistochemistry and qRT-PCR techniques revealed that carrageenan injection increased significantly (*p* < 0.05) the activity and expression of COX-2 and iNOS in the inflamed paw tissue. Furthermore, PGE2 and NO levels in paw edema skin were elevated significantly as compared to their corresponding control group (Figures 3, 5, and 6). In contrast, COX-2 and iNOS expressions and their products were significantly decreased following DADS administration as compared to the carrageenan-injected mice; reflecting its potent anti-inflammatory activity against carrageenan-induced acute inflammatory response. Diclofenac-treated mice showed similar results as compared to the carrageenan-injected group.

### 4.7. Effects of Diallyl Disulfide on MCP-1 Level and mRNA Expression and MPO Activity

**Figure 7:**
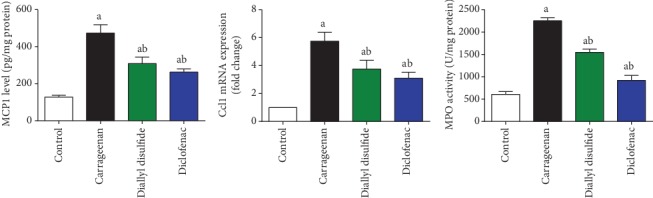
Effect of diallyl disulfide (DADS, 100 mg/kg) or diclofenac (20 mg/kg) on levels and mRNA expression of monocyte chemoattractant protein-1 (MCP-1, *Ccl1*) and myeloperoxidase (MPO) activity. ELISA and spectrophotometric data are represented as mean ± SD (*n* = 7). qRT-PCR data are presented as mean ± SD of triplicate assays and referenced to *Gapdh*. ^a^*p* < 0.05 indicates a significant change versus the control group; ^b^*p* < 0.05 indicates a significant change versus the carrageenan-injected mice.

During inflammatory conditions, MCP-1 recruits and regulates the migration and infiltration of monocytes and other leukocytes into the injured site. Meanwhile, MPO activity reflects the neutrophil migration to in the inflamed tissue. Carrageenan injection showed a significant (*p* < 0.05) increase in MCP-1 level and mRNA expression and MPO activity in inflamed hind limb as compared to the untreated group (Figure 7). In contrast, DADS-administered mice exhibited a significant decrease in the level and expression of MCP-1 and MPO activity as compared to the carrageenan-treated group, reflecting its anti-inflammatory effect through inhibiting neutrophil migration and infiltration and-macrophage derived chemokines at the inflamed foci. Diclofenac-administered mice showed a significant decrease in the level and mRNA expression of MCP-1 and MPO activity in the inflamed tissue as compared to the carrageenan-injected group.

### 4.8. Effects of Diallyl Disulfide on Carrageenan-Induced Oxidative Stress

**Figure 8:**
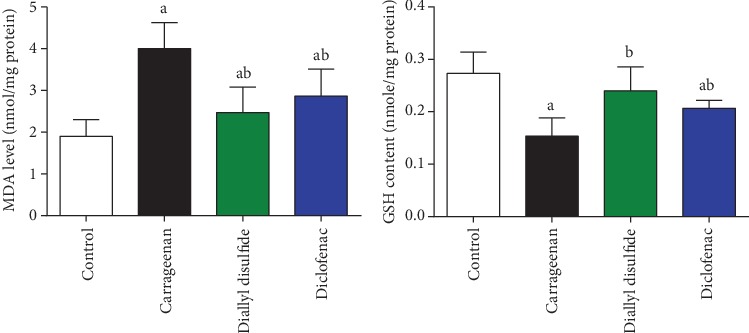
Effect of diallyl disulfide (DADS, 100 mg/kg) or diclofenac (20 mg/kg) on oxidative stress marker malondialdehyde (MDA) and glutathione (GSH) in carrageenan-induced paw edema in mice. Data are represented as mean ± SD (*n* = 7). ^a^*p* < 0.05 indicates a significant change versus the control group; ^b^*p* < 0.05 indicates a significant change versus the carrageenan-injected mice.

To evaluate the change in the oxidative status in the inflamed skin tissue, levels of MDA and GSH were examined (Figure 8). The injected mice with carrageenan elicited a significant increase in the level of MDA and decreased GSH content when compared to the control mice. Oral administration of DADS and diclofenac inhibited significantly the oxidant (MDA) and increases the antioxidant (GSH) in the paw skin following carrageenan injection.

## 5. Discussion

Inflammatory reactions are characterized by vascular hyperpermeability and cellular changes including enhancing ROS overproduction and the activation of inflammatory mediators in order to reduce the cellular injury [18]. The current treatments for the inflammatory disorders are coupled with undesirable side effects. Hence, development of new naturally derived alternative agents for the treatment of inflammation and the related disorders, with minimum or negligible side effects, is mandatory. The carrageenan-induced paw edema is a well-established acute inflammation experimental model and has been used to estimate the potential anti-inflammatory impact of natural compounds. Indeed, carrageenan induction in the mice hind paw triggered intense inflammatory events characterized by oversecretion of numerous inflammatory mediators, temporary vasoconstriction, and vasodilatation, resulting in the development of edema and enlarged its size which peaked after 8 h [2, 18]. In the present study, DADS was experimented for its potential anti-inflammatory and antioxidant effects against carrageenan-induced paw edema in mice. Moreover, carrageenan injection elevated significantly the CRP level. It has been demonstrated that inflammatory response in the injured tissue following endogenous or external stimuli like carrageenan triggers production of proinflammatory proteins including CRP [19]. DADS administration exerted potent anti-inflammatory impact against carrageenan injection through decreasing significantly the volume of developed paw edema and CRP level after 8 h.

Carrageenan-induced acute inflammation is coupled with the progression of biphasic inflammatory responses in the paw tissue. The early phase include mainly the production of histamine, serotonin, and bradykinin, whereas the late phase includes the production of neutrophil-derived free radicals, proinflammatory cytokines, NO synthesis, neutrophil infiltration, and COX-2 activation and subsequently prostaglandin production [20]. In the current study and after 8 h, carrageenan injection triggered the activation and production of TNF-*α*, IL-1*β*, IL-2, NO, iNOS, COX-2, and PGE2 in the injured skin tissues. Cross-talk between different inflammatory mediators has been confirmed following carrageenan injection. TNF-*α*, IL-1*β*, and IL-2 are proinflammatory cytokines produced by immune cells (macrophages and monocytes) and nonimmune cells (fibroblasts and endothelial cells) in response to inflammation and cellular injury [21]. TNF-*α* enhances IL-1*β*, kinin, and leukotriene production, which regulates and controls the long-lasting nociceptive reaction. IL-*1β* increases the expression of adhesion molecules on endothelial cells to enable transmigration of leukocytes and is coupled with hyperalgesia [22]. TNF-*α* and IL-1*β* are able to stimulate PGE2 formation through activating COX-2. In addition, TNF-*α* is known to activate iNOS expression and enhance neutrophil migration to the injured foci in response to an inflammatory stimuli [20]. Moreover, the upregulated iNOS has been suggested to play a crucial role in the development of inflammatory responses through enhancing NO formation. NO is a ubiquitous intercellular molecule regulating several physiological functions. However, at high concentrations, NO react directly with superoxide anions to produce peroxynitrite radicals, resulting in cellular oxidative and inflammatory events [23]. On the other hand, NO has been suggested to liberate arachidonic acid from the cell membrane, which in turn activates COX-2. Different observations suggest that downregulating gene expression of macrophage-related proinflammatory mediators and inhibiting COX-2 and iNOS activities exhibited a therapeutic effect against some inflammatory conditions [6]. NF-*κ*B is a transcriptional factor, which regulates and controls the production and regulation of a variety of proinflammatory mediators [24]. Here, NF-*κ*B was found to be overexpressed in response to carrageenan injection. This may explain the increased levels and mRNA expression of TNF-*α*, IL-1*β*, NO, iNOS, and COX-2 in the inflamed paw tissue. NF-*κ*B activation following carrageenan injection has been attributed to ROS overproduction in the damaged skin tissue [23].

Carrageenan injection decreased IL-10 level, upregulated the level and mRNA expression of MCP-1, and activated MPO in the paw tissue. IL-10 is an anti-inflammatory cytokine produced by T-cells and monocytes/macrophages and is known to suppress the secretion of proinflammatory cytokines [25, 26]. The imbalance between pro- and anti-inflammatory cytokines is associated with the progression of local inflammatory response. Corsini et al. [27] reported a significant decrease in IL-10 levels following carrageenan injection. The authors attributed this effect to dysregulation of cAMP signal-transduction pathway. The decreased IL-10 production results in a continual neutrophil infiltration, as evaluated by the measurement of levels of the specific granulocyte enzyme MPO, and in more severe tissue damage, as evaluated by studying histological alterations [27]. MCP-1 is a chemokine which regulates the migration and infiltration of monocytes/macrophages from the blood stream across the vascular endothelium in response to inflammation. MCP-1 was found to be elevated upon carrageenan injection in a pleural inflammatory model, reflecting the infiltration of innate immune cells at damaged tissue [28]. Restoring levels of anti-inflammatory cytokines and downregulating the gene expression of macrophage-derived chemokines show remedial effects in some inflammatory conditions. The increased endogenous IL-10 elicits an anti-inflammatory activity during acute inflammation and tissue damage coupled with carrageenan-induced pleurisy in rats [27].

In this study, DADS administration decreased and downregulated TNF-*α*, IL-1*β*, NO, iNOS, COX-2, and PGE2 which confirm its anti-inflammatory activity against carrageenan-induced acute inflammatory response in paw tissue. This effect was further confirmed through increasing IL-10 level, deactivating NF-*κ*B inhibiting migration and infiltration of leucocytes and macrophage-derived chemokines, as indicated by the downregulated MCP-1 level and expression and suppressed MPO activity. We assume that the decreased NO may be due to the downregulation of iNOS and COX-2, while the reduced proinflammatory mediators may be due to the inactivation of NF-*κ*B. The anti-inflammatory activity of DADS was reported in different experimental designs. Chen et al. [29] revealed that DADS supplementation may be used as an alternative therapy for the treatment of rheumatoid arthritis via reducing the paw volume and suppressing the production of proinflammatory mediators. DADS was found to decrease mRNA and protein levels of iNOS upon carbon tetrachloride exposure [30]. Additionally, DADS administration protected the gastric mucosa against alcohol-induced acute inflammation via reducing TNF-*α* and IL-6 along with iNOS downregulation [31]. Furthermore, DADS showed anti-inflammatory activity against cerulein-mediated pancreatic inflammation and its associated pulmonary deficits through inhibiting the generation of TNF-*α*, cystathionine-*γ*-lyase, preprotachykinin A, neurokinin-1-receptor expression, and hydrogen sulfide along with inhibiting I-*κ*B degradation and NF-*κ*B expression and translocation in the pancreatic and pulmonary tissue [32]. In the same context, DADS exerted an anti-inflammatory effect through quenching ROS and inhibiting the NF-*κ*B signaling pathway in human Barrett's epithelial cells [33]. DADS was reported to prevent the development of colorectal tumor by blocking inflammation, a process largely involving GSK-3*β* suppression and the inhibition of NF-*κ*B nuclear translocation [34]. Earlier study demonstrated that DADS administration suppressed significantly NO and PGE2 associated with the downexpression of iNOS and COX-2 and decreased the level and expression of TNF-*α*, IL-1*β*, and MCP-1 in lipopolysaccharide- (LPS-) stimulated BV2 microglia [35].

Carrageenan injection potentiated oxidative challenge in the skin tissue as presented by increasing MDA and decreasing GSH content. It has been reported that carrageenan is able to enhance free radical production including superoxide anions, hydrogen peroxides, nitric oxide, and its cytotoxic derivative [23, 36]. In pathophysiological conditions, MDA formation reflects the oxidation of membrane lipids following free radical production and has been found to be associated with inflammatory and oxidative responses [15]. GSH represents the first antioxidant defense line against free radicals and its consumption is correlated with the development of lipid peroxidation and further oxidative stress. Antioxidant molecules that are able to inhibit lipid peroxidation and downregulation of *Nos2* may be used to treat or alleviate carrageenan-induced paw edema. DADS administration ameliorated successfully the redox homeostasis in response to carrageenan-induced oxidative damage in the skin tissue, as evidenced by the increased GSH content and inhibited MDA. In accordance with these data, Liu et al. [9] demonstrated that DADS inhibited the oxidative damage in an emphysema model in rodents through inhibiting lipid peroxidation and enhancing the endogenous antioxidant defense molecules. Furthermore, DADS prevented the progression of oxidative insults in the liver tissue following ethanol intoxication through quenching free radicals and enhancing Nrf2/HO-1 activity [37].

## 6. Conclusion

Collectively, we demonstrated that DADS administration inhibited the paw edema development by acting at both phases of the carrageenan-induced inflammation. DADS elicited its anti-inflammatory activity by suppressing blood CRP level, proinflammatory cytokines release (TNF-*α*, IL-1*β*, and IL-2), and inhibiting iNOS and COX-2 expressions and their products (NO and PGE2) through blocking NF-*κ*B activation. DADS also inhibited migration and infiltration of leucocytes and macrophage-derived chemokines at the injured site. Furthermore, we revealed that DADS could improve the injured skin tissue following carrageenan injection through its antioxidative activity. However, further studies are required to ascertain the molecular anti-inflammatory mechanism of DADS using different experimental studies.

## Figures and Tables

**Table 1:** Primer sequences of genes analyzed in real time PCR.

Name	Accession number	Sense (5′—3′)	Antisense (5′—3′)
Gapdh	NM_001289726.1	CCCATCACCATCTTCCAGGAGC	CCAGTGAGCTTCCCGTTCAGC
Ccl2	NM_011333.3	GCAGCAGGTGTCCCAAAGAA	ATTTACGGGTCAACTTCACATTCAA
Il2	NM_008366.3	TGAGTCAGCAACTGTGGTGG	GCCCTTGGGGCTTACAAAAAG
Il10	NM_010548.2	ATAACTGCACCCACTTCCCA	GGGCATCACTTCTACCAGGT
Il1b	NM_008361.4	CCTTCCAGGATGAGGACATGA	TGAGTCACAGAGGATGGGCTC
Nos2	NM_001313922.1	CGAAACGCTTCACTTCCAA	TGAGCCTATATTGCTGTGGCT
Ptgs2	NM_011198.4	CAGACAACATAAACTGCGCCTT	GATACACCTCTCCACCAATGACC
Tnf*α*	NM_001278601.1	ACCCTCACACTCACAAACCA	ACCCTGAGCCATAATCCCCT

The abbreviations of the genes: Gapdh: glyceraldehyde-3-phosphate dehydrogenase; Ptgs2: prostaglandin-endoperoxide synthase 2 (COX-2); Nos2: nitric oxide synthase 2, inducible; Il1b: interleukin 1 beta; *Tnfα*: tumor necrosis factor alpha; Ccl2: chemokine (C-C motif) ligand 2 (MCP-1).

## Data Availability

The data used to support the findings of this study are included within the article.

## Supplementary Materials

Supplementary Materials Effect of diallyl disulfide (DADS, 100 mg/kg) or diclofenac (20 mg/kg) on paw skin NF-*κ*B and iNOS expression intensity detected by immunohistochemistry following carrageenan injection-induced paw edema in mice.
