# Supplementary material for: Diallyl Disulfide Suppresses Inflammatory and Oxidative Machineries following Carrageenan Injection-Induced Paw Edema in Mice
Source: Mediators Inflamm. 2020 Apr 15;2020:8508906. doi: 10.1155/2020/8508906 (PMC7180418; doi:10.1155/2020/8508906)
Supplement: Supplementary Materials — Effect of diallyl disulfide (DADS, 100 mg/kg) or diclofenac (20 mg/kg) on paw skin NF-κB and iNOS expression intensity detected by immunohistochemistry following carrageenan injection-induced paw edema in mice. [file 8508906.f1.pdf]

Supplementary data

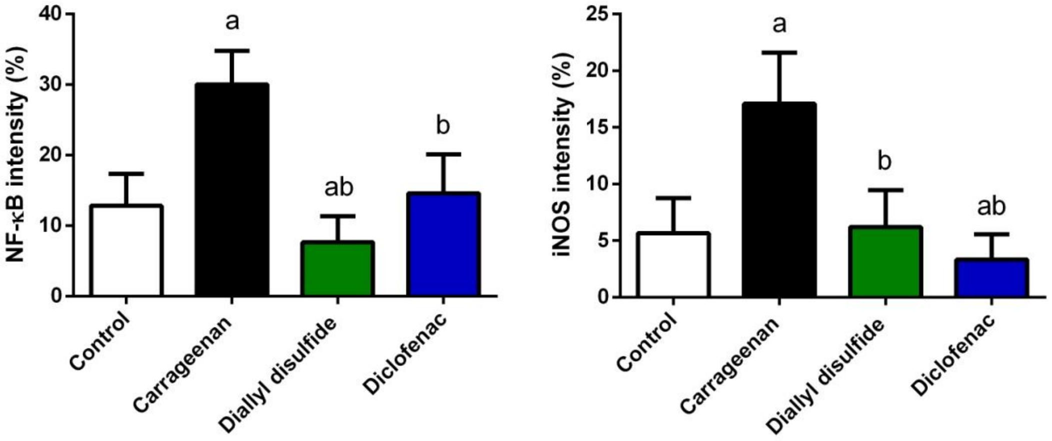

Fig. S1: Effect of diallyl disulfide (DADS, 100 mg/kg) or diclofenac (20 mg/kg) on paw skin NF-κB and iNOS expression intensity detected by immunohistochemistry following carrageenan injection-induced paw edema in mice.
